# Eddy-Current Sensors with Asymmetrical Point Spread Function

**DOI:** 10.3390/s16101642

**Published:** 2016-10-04

**Authors:** Janusz Gajda, Marek Stencel

**Affiliations:** Department of Measurement and Electronics, Faculty of Electrical Engineering, Automatics, Computer Science and Biomedical Engineering, AGH University of Science and Technology, al. A. Mickiewicza 30, 30-059 Krakow, Poland; jgajda@agh.edu.pl

**Keywords:** point spread function, deconvolution, inverse problem, eddy-current, magnetic signature, spatial scanning

## Abstract

This paper concerns a special type of eddy-current sensor in the form of inductive loops. Such sensors are applied in the measuring systems classifying road vehicles. They usually have a rectangular shape with dimensions of 1 × 2 m, and are installed under the surface of the traffic lane.

The wide Point Spread Function (PSF) of such sensors causes the information on chassis geometry, contained in the measurement signal, to be strongly averaged. This significantly limits the effectiveness of the vehicle classification. Restoration of the chassis shape, by solving the inverse problem (deconvolution), is also difficult due to the fact that it is ill-conditioned.

An original approach to solving this problem is presented in this paper. It is a hardware-based solution and involves the use of inductive loops with an asymmetrical PSF. Laboratory experiments and simulation tests, conducted with models of an inductive loop, confirmed the effectiveness of the proposed solution. In this case, the principle applies that the higher the level of sensor spatial asymmetry, the greater the effectiveness of the deconvolution algorithm.

## 1. Introduction

The spatial aperture of eddy-current sensors, used in the measurement of road traffic, causes the blurring of the signal which represents the shape of the object. This causes a distortion in information on the actual shape of the object. The greater the width of the aperture, the stronger its averaging properties. Averaging of the signal, however, results in a loss of information on the details of the geometry of the object studied. In many applications, inductive loops are used to scan the shape of the chassis of vehicles. The measuring signal obtained in this way is known as the magnetic signature of the vehicle, and is commonly used for its classification [1,2,3,4]. Loss of information on details of the geometry of vehicles therefore causes a loss of resolution in their classification.

The specific design of inductive loops used in the measurement of traffic parameters makes their properties significantly different from those of conventional eddy-current sensors used in non-destructive testing (NDT). The main differences concern: the dimensions of the sensor, which in the case of the inductive loop are in the order of meters, the spatial extent of the generated magnetic field to approx. 0.8 m and a spatial resolution of 0.2 m. Identical however, is the fundamental purpose of the research work conducted with both groups of sensors. It is the increasing effectiveness of the classification of the measured objects. Studies described in this work are the confirmation of this thesis [5]. It proposes the use of PCA (Principal Component Analysis) for increasing the efficiency of subsurface crack measurement. The success, however, is conditioned by the amount of data contained in the raw signal of the sensor. The solution described in this paper allows an increase of the amount of data contained in a signal which can then be further processed (e.g., PCA, neural network, wavelet analysis etc.), resulting in the correct classification of the object.

**Figure 1:**
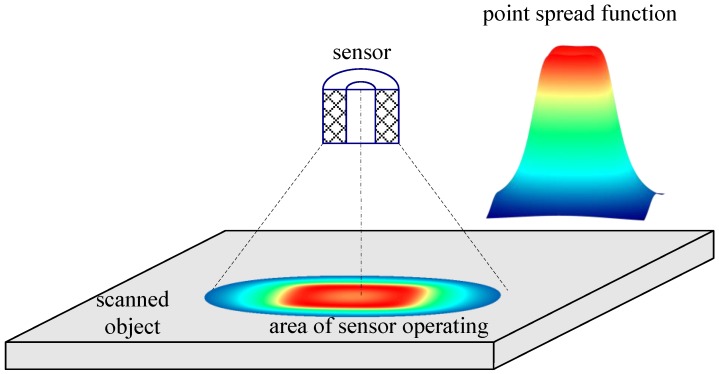
Illustration of the spatial aperture of an eddy-current sensor.

Blurring of the magnetic signature is caused by spatial averaging properties of the eddy current sensor, whose output signal depends on the total metal surface of the object affected by the alternating magnetic field generated by the sensor. This phenomenon is illustrated in the diagram presented in Figure 1. It is present not only in the sensors described above, but also in all types of sensors used in the process of spatial scanning [6].

If the vehicle moves over the sensor along a straight line, a one-dimensional signal is created at the output of the sensor, which is a function of the time or distance covered by the vehicle, including averaged information on the shape of the chassis. Both the surface width and length of the vehicle are averaged.

There is a large number of studies which describe this process by means of a convolution model [7,8,9]. The function, which is responsible for the averaging process, is interpreted as a Point Spread Function (PSF) of the sensor. The output signal of the sensor is interpreted as a convolution [10] of the function describing the shape of the object and the PSF of the sensor, and is described by Equation (1).

(1) $$y\left(l\right)=\int_{-\infty }^{+\infty }h\left(l-\lambda \right)x\left(\lambda \right)d\lambda$$

In which: *h*(*λ*) is the PSF, *x*(*λ*) is a function describing the geometrical shape of the object, and *y*(*l*) is the output signal of the sensor, which is the averaged (spatially blurred) result of the scan.

**Figure 2:**
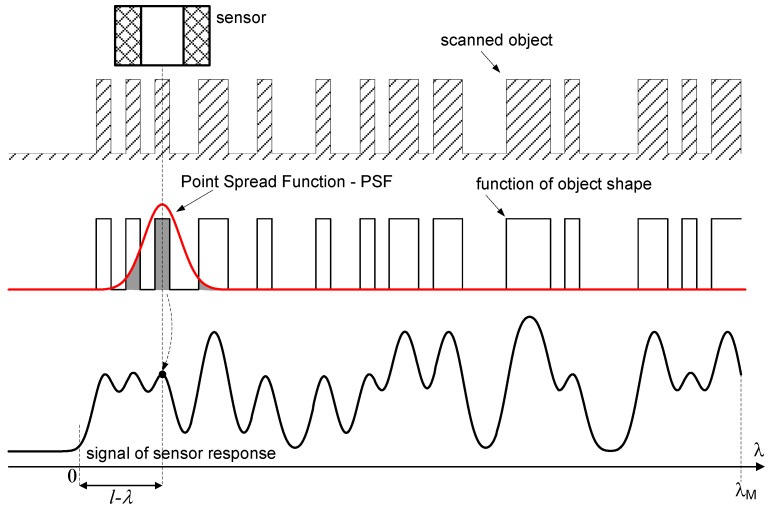
One-dimensional object scanning with a sensor with aperture.

A graphic illustration of the convolution operation described by Equation (1), relevant to a one-dimensional case, is presented in Figure 2.

## 2. Convolution Matrix

Taking into account the limited dimensions of the object, Equation (1) looks as follows Equation (2):

(2) $$y\left(l\right)=\int_{\lambda =0}^{\lambda_{M}}h\left(l-\lambda \right)\;x\left(\lambda \right)d\lambda$$

in which: $0\leq \lambda \leq \lambda_{M\;}$— the dimension of the object.

Following the digitisation of the distance $\lambda_{M}=\Delta l\times M$, *l = Δl × n*, λ=Δl×m, in which *Δl* is the quantum of the distance, a convolution form is expressed by the Equation (3).

(3) $$\frac{y\left(n\right)}{\Delta l}=y_{n}=\sum_{m=0}^{M}h_{n-m}x_{m}\;\mathrm{for}\;0\leq m\leq M$$

By applying a matrix form, Equation (3) can be presented as Equation (4)

(4) $$\left[\begin{array}{c} y_{0}\\ y_{1}\\ y_{2}\\ \vdots \\ y_{M} \end{array}\right]=\left[\begin{array}{ccccc} h_{0} & h_{-1} & h_{-2} & \cdots & h_{-M}\\ h_{1} & h_{0} & h_{-1} & \ddots & \vdots \\ h_{2} & h_{1} & h_{0} & \ddots & \vdots \\ \vdots & \ddots & \ddots & \ddots & \vdots \\ h_{M} & \cdots & \cdots & \cdots & h_{0} \end{array}\right]\left[\begin{array}{c} x_{0}\\ x_{1}\\ x_{2}\\ x_{M} \end{array}\right]$$

or symbolically as Equation (5)

(5) Y=H⋅X,

in which *h_−M_*.....*h*_0_, *h*_1_ ... *h_M_*, are spatial samples of the PSF, *y*_0_ ÷ *y_M_*, are samples of the output signal, while *x*_0_ ÷ *x_M_*, are samples of the signal of the object’s shape.

The size of the ***H*** matrix depends on the length of the observed signals at the beginning and end of the sensor, and is equal to the length of the vector signal ***X***.

The averaging effect of the sensor depends on the elements of the ***H*** matrix. This effect may be eliminated or at least limited by calculating, from Equation (5), the signal of the shape of the object ***X***, assuming that the ***Y*** signal and PSF of the sensor used are known.

An estimate of the ***X*** vector can be obtained from Equation (6). This is a reverse operation to the convolution operation and is known as deconvolution:

(6) $$X_{OD}=H^{-1}\cdot Y$$

in which the ***H*^−1^** matrix is the inverse matrix of ***H***, ***X_OD_*** is the estimate of the signal of the shape of ***X***.

Equation (6) defines the inverse problem, which, due to the singularity of the ***H*** matrix or low value of its determinant, is ill-conditioned. This is the cause of substantial numerical errors which distort the solution of this problem. This is why one speaks of an estimate of the signal of the shape and not of its precise calculation. The precision of this estimate depends on the effectiveness of the numerical deconvolution algorithm applied.

The form of the ***H*** matrix depends on the PSF of the sensor, which in turn depends on its construction. It is always, however, a matrix with a Toeplitz structure [11,12]. In the most general case, it takes the form of Equation (7).

(7) $$H=\left[\begin{array}{ccccc} h_{0} & h_{-1} & h_{-2} & \cdots & h_{-M}\\ h_{1} & h_{0} & h_{-1} & \ddots & \vdots \\ h_{2} & h_{1} & h_{0} & \ddots & \vdots \\ \ddots & \ddots & \ddots & \ddots & \vdots \\ h_{M} & \cdots & \cdots & \cdots & h_{0} \end{array}\right]$$

Depending on the PSF shape of the sensor, the form of the ***H*** matrix can take slightly different forms. For causal systems described for time-domain, this is a lower triangular matrix. For systems described in terms of spatial coordinates, as in the case of an inductive loop, this is in the form of Equation (7). If the PSF is relatively narrow with regard to the range of the input signal ***X***, the ***H*** matrix becomes a diagonal. Outside of the band around the main diagonal, the values of the matrix elements are equal to zero. Sensors described by this type of matrix are characterised by high spatial resolution, and causes only small distortions in the signal of the shape of the object. For systems with a relatively wide PSF, this matrix takes the form of a full matrix. Such sensors significantly average the shape signal, blurring geometric details of the object. In the case of spatial systems, ***H*** matrices, either band or full, may be either symmetrical or asymmetrical. This symmetry, or its absence, results from the PSF shape, which is in turn associated with the construction of the sensor itself. For sensors with a symmetrical PSF, the ***H*** matrix is also symmetrical, while in the opposite case it is asymmetrical. A set of different forms of the H matrix is presented in Table 1.

Equation (6) describes the simplest but also least precise deconvolution algorithm, resulting directly from the convolution Equation (5). In practice, other algorithms are used which minimise the influence of distortions that appear in the measuring signals [7,8,13,14]. One of these is the least squares algorithm (LS) [1,15,16], described by Equation (8).

(8) $$X_{OD}={\left(H^{T}H\right)}^{-1}H^{T}\cdot Y$$

In order to improve the conditioning of the inverse problem, described by Equation (8), additional regularisation is also often used [17,18].

The necessity of regularization results from the need to minimize the numerical errors of the matrix invertion. The determinant of the symmetric matrix equals zero and inverting of such a matrix is impossible. The proposed method de-symmetrizes the convolution matrix using the sensors with an asymmetric PSF. As a result, the determinant value of the inverted matrix’s increases and the numerical errors are smaller.

**Figure 3:**
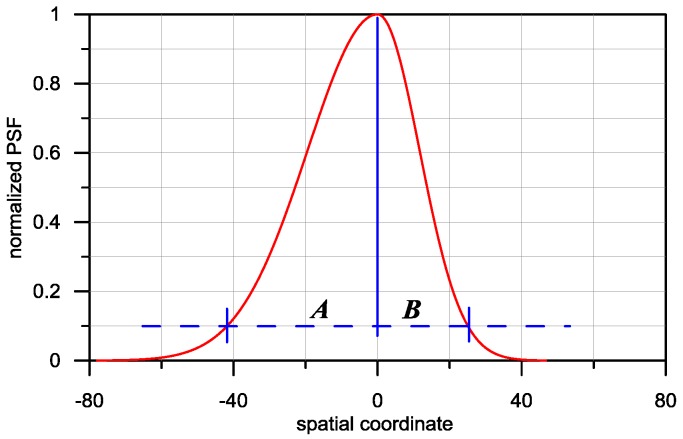
Illustration of a method of PSF (Point Spread Function) asymmetry estimation.

In order to obtain a quantitative description of the relation between the degree of PSF asymmetry and the value of the ***H^T^H*** matrix determinant, a factor of PSF asymmetry is introduced in the form of Equation (9). Its width is equal to the sum of A + B.

(9) $$W=\frac{2A}{A+B}$$

in which: A and B are the length of the relevant sections measured at 10% of the maximum normalised PSF value, as indicated in Figure 3.

For a symmetrical PSF *W* = 1.0, whereas *Wmax* = 2.0 for maximum asymmetry, where B = 0, and A is a number other than 0.

## 3. Simulation Studies of Asymmetrical PSF

**Figure 4:**
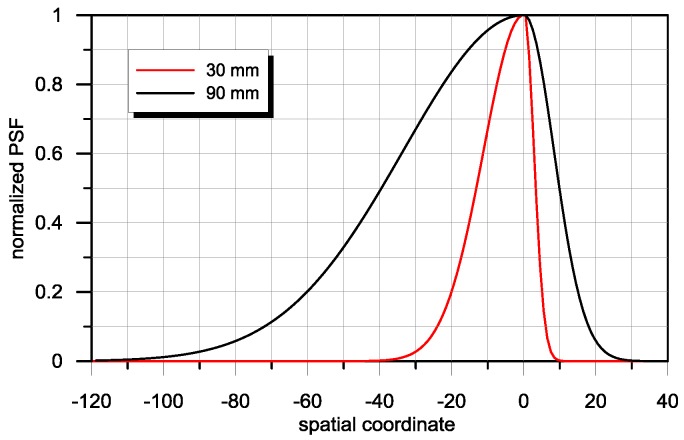
Examples of two PSF with a width of 30 mm and 90 mm and identical asymmetry factor values *W* = 1.6.

In order to quantitatively study the influence of the PSF asymmetry of the sensor on the quality of restoration of the object shape signal, simulation studies were conducted. As a PSF model for the sensor, a spline function was used, composed of two normalised Gauss curves with different factor values. For a defined value of *W*, the PSF may be both wide, as in the case of a full, asymmetrical **H** matrix; or narrow, as in the case of an asymmetrical band matrix. Examples of a PSF with uniform asymmetry *W* = 1.6 and different width is presented in Figure 4.

**Figure 5:**
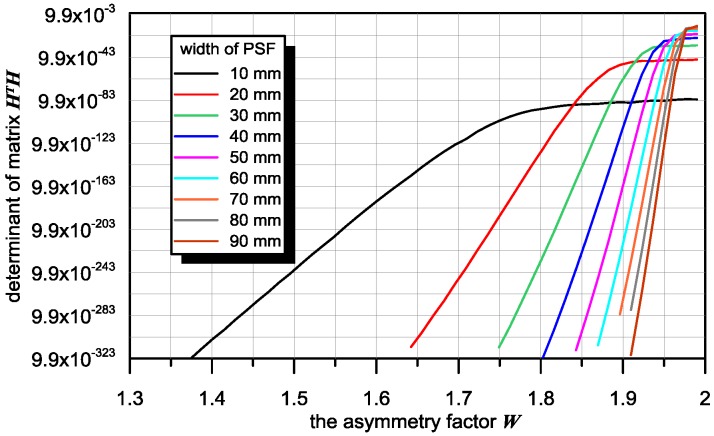
Dependence of the convolution matrix determinant on the asymmetry factor W.

The results of simulation studies presented in Figure 5 confirm that the value of the H matrix determinant is strongly dependent on PSF asymmetry. Its value also depends on PSF width.

The simulations conducted were intended to define the relation between the degree of PSF asymmetry and the restoration error Equation (10)

(10) $$D=\sqrt{\frac{\sum_{1}^{N}{\left(X_{OD}-X\right)}^{2}}{\sum_{1}^{N}X^{2}}}$$

in which: ***X***—input shape signal, ***X_OD_***—estimate of signal ***X***—result of deconvolution.

**Figure 6:**
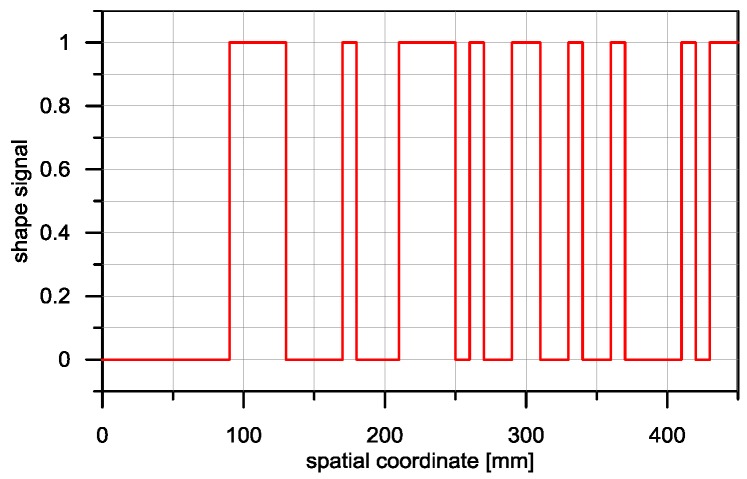
Arbitrary input signal X used in simulation studies.

In the simulations conducted, for the input signal ***X***, a pseudorandom binary signal was used, a fragment of which is presented in Figure 6.

**Figure 7:**
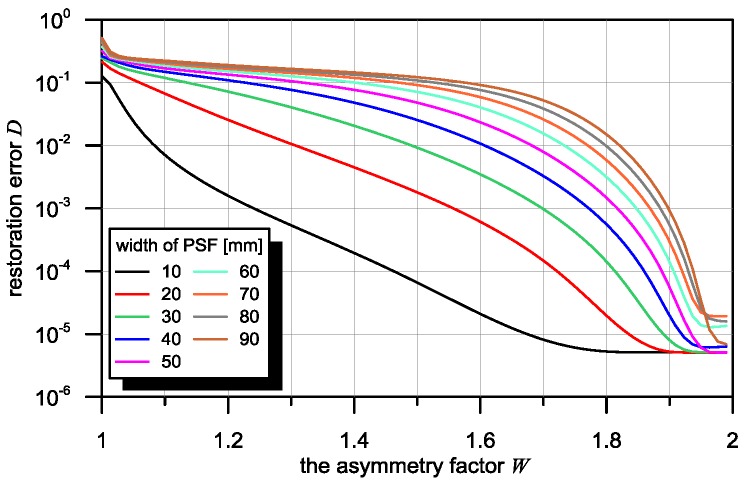
Dependence of restoration error D on asymmetry factor W in the full variability range for various PSF widths of the sensor (from 10 mm to 90 mm). Ideal case: without distortion in the measuring signal.

The dependence of the restoration error D on the asymmetry factor W for PSF of various widths was obtained for Algorithm (8) and presented in Figure 7. A change in the asymmetry factor of the sensor in range of input signal ***X***, while maintaining a stable PSF width, causes a change in the value of the restoration error by several degrees of magnitude. Simultaneously, a relation can be observed involving the strong dependence of the restoration error on the PSF width of the sensor with a constant asymmetry factor value W. This is understandable, as sensors with smaller PSF widths have better spatial resolution of the magnetic signature. In such a case, the object signal shape is weakly averaged.

**Figure 8:**
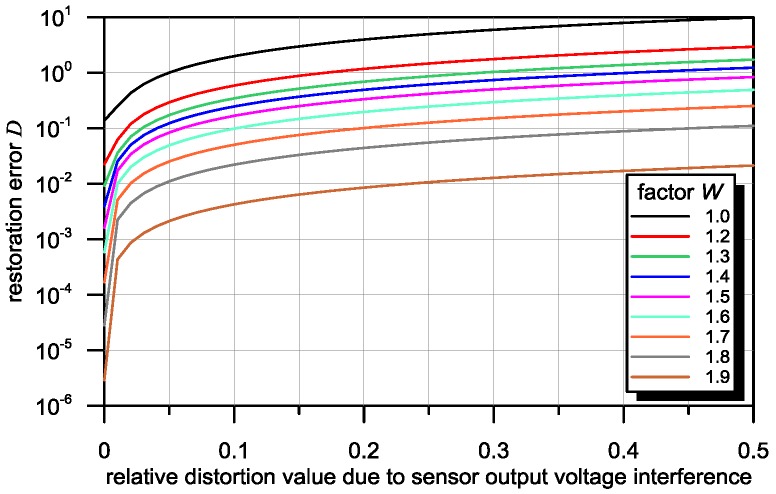
Dependence of restoration error D on distortion level for sensors with various asymmetry factors (W: 1.0–1.9) and PSF widths of 40 mm.

In Figure 8, characteristics illustrating the influence of additive random distortions included in the output signal Y of the sensor on the restoration error D, are presented. These characteristics are relevant to PSF with a width of 40 mm and with various asymmetry factors. As a measure of the strength of these distortions, their relative value with respect to the maximum input signal X is applied. The greater the level of distortion, the greater the required asymmetry of the sensor is, in order to obtain the required value of the restoration error D.

**Figure 9:**
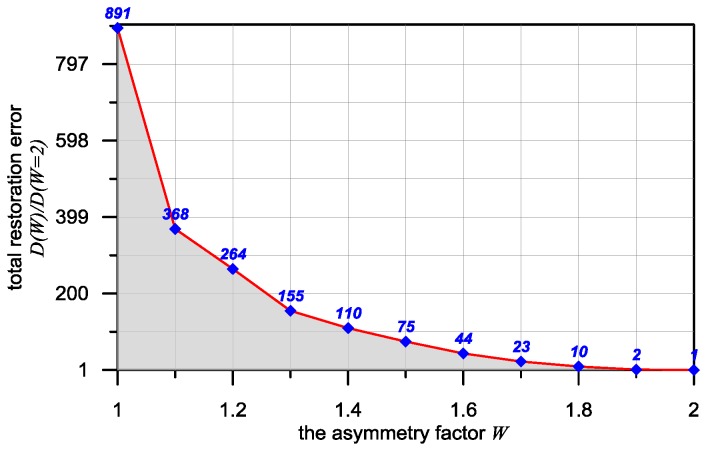
Value of multiples restoration error as a function of the asymmetry factor W in the presence of additive random distortions.

In Figure 9, a characteristic which makes it possible to assess the benefits of using sensors with asymmetrical PSF is presented. It shows the relative value of the restoration error D(W) of the estimate *X_OD_*, correlated with the value of this error for W = 2.0, that is D(W = 2.0). The characteristic was indicated in the presence of additive random distortions included in the signal Y with a relative standard deviation equal to 0.5%.

All simulations were made in the Matlab R2009b software package of Mathworks in the double precision format [19].

## 4. Experimental Studies

**Figure 10:**
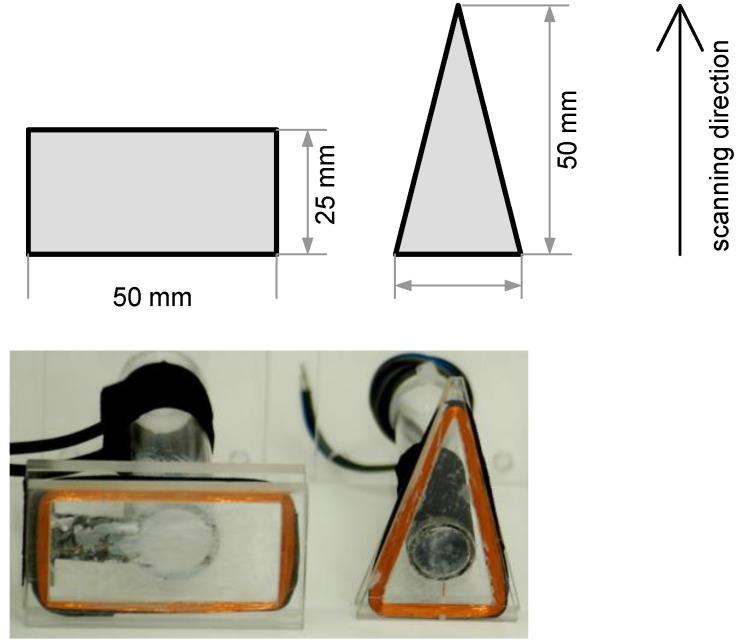
Rectangular and triangular inductive loop models.

In order to experimentally confirm the results of the simulation studies, two inductive loop models with shapes and dimensions presented in Figure 10 were built. (rectangular: turns of coil = 250, resistance = 42.3 Ω, inductance = 5.6 mH, triangular: turns of coil = 270, resistance = 39.2 Ω, inductance = 4.74 mH).

**Figure 11:**
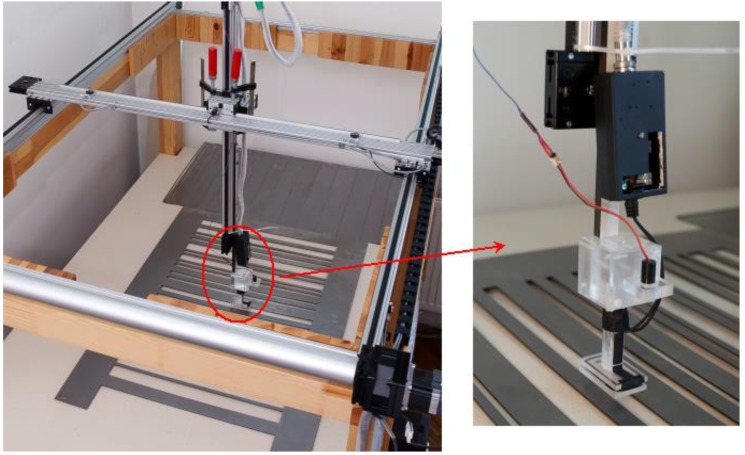
The scanner used during the measurement with an installed eddy-current sensor and a steel sheet.

Figure 11 shows the experiment setup. The applied scanner ensures a precise displacement of the eddy-current sensor over the scanned object and allows measurement of the traveled distance, as well as the sensor output signal. Maximum length of the scanned path is 1000 mm, and attainable measurement resolution is 0.1 mm. The method applied by authors for determining the sensor’s PSF employs a pseudorandom binary (PRB) signal generated by the cut out steel sheet as the input signal exciting the identified ILD (Inductive Loop Detector). If DX means the bit width, the logic state “1” corresponds to the presence of a steel sheet strip, and the state “0” corresponds to an opening cut out in the steel sheet. The signal length is defined by the number of bits N0.

During experiments, a signal was used with the number of bits N0=500 and DX=10 mm, that means the total geometrical length of a sheet in which the signal was cut out is 5000 mm. The sheet was divided into seven separate sections with an additionally repeated ending sequence of bits from the leading section and initial sequence of bits from the section following the currently scanned portion of the PRB signal, as shown in Figure 3. The tested detector is shifted at a fixed height along consecutive sections. Thus the detector “sees” a continuous PRB signal despite the fact that the experiment setup length is much shorter than the signal geometrical length whereas, due to the large width of the sheet, its solid edges that function only as a structural component are not “seen” by the detector. During the experiment, the sensors have been supplied with the frequency of 10 kHz. The measured signals have been processed using the correlation method. Seeking the sensors’ PSF is determined by the numerical solution of the Winer-Hopf equation.

**Figure 12:**
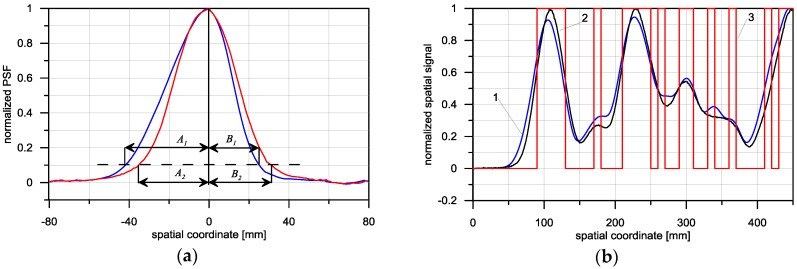
Experimentally obtained characteristics for inductive loop models (**a**) PSF for triangular (1) and rectangular (2) detector; (**b**) output signals corresponding to shape signal in the form of PRBS (Pseudo Random Binary Signal): 1—Output signal of the triangular detector, 2—Output signal of the rectangular detector, 3—Shape signal—Input signal.

The asymmetrical shape of the triangular detector with regard to the direction of scanning should result in an asymmetrical PSF in this detector. These assumptions are confirmed by the characteristics presented in Figure 12. In Figure 12a, the PSF of both detectors is presented. For both of them, these were established experimentally [16].

The *W* factor in the case of a triangular detector has a value of *W* = 1.25, while for the rectangular detector that value is *W* = 1.05. The triangular detector is therefore indeed an asymmetrical detector. The slight asymmetry appearing in the case of the rectangular detector may have been caused by the uneven placement of the electromagnetic coils or by errors in the PSF identification. The PSF width of both detectors is comparable, for the rectangular detector amounting to *A + B* = 66.8 mm, and for the triangular detector *A + B* = 66.2 mm.

For both detectors compared, responses to stimulation by the PRBS signal were registered. The output signals obtained from both sensors are very similar. Due to the comparable PSF width of both sensors, they average input signals in a similar way, causing the loss of part of the information regarding the shape of the object measured (the cut steel sheet). The difference between the output signal of the detector and the input signal, measured with regard to error *D*, is *D* = 0.59 for the triangular detector, and *D* = 0.58 for the rectangular detector.

In an ideal case, when the PSF of the detector is precisely known and the deconvolution algorithm has been performed perfectly, the restoration input signal should precisely describe the shape of the object, thus allowing it to be perfectly recognised. In reality, neither of these conditions is fulfilled, and the result of deconvolution, only to a greater or lesser extent, reflects the input signal. It is only an estimate. The sensor’s construction methods undertaken and algorithms used are intended to improve the precision of this solution.

**Figure 13:**
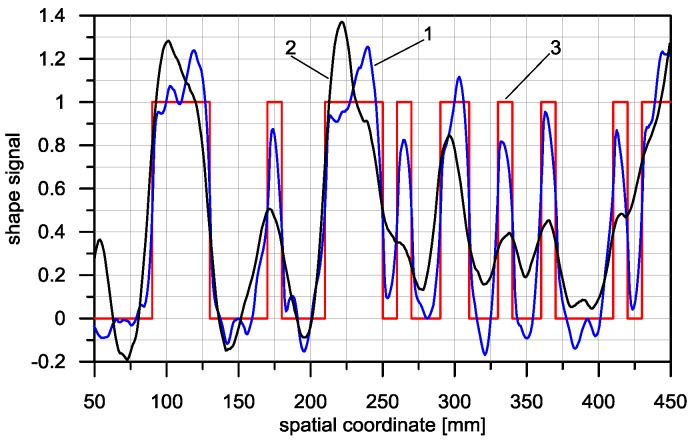
Deconvolution result using the LS (Least Square) algorithm. 1—For the triangular detector, 2—For the rectangular detector, 3—Shape of scanned object.

In Figure 13, the deconvolution results obtained for the triangular detector and for the rectangular detector using the LS algorithm are presented. In the case of the triangular detector, the restoration signal includes considerably more details and more precisely describes the shape of the scanned object when compared with the result of the rectangular detector. The restoration error, accepted as a measure of the difference between the deconvolution result *X_OD_* and the shape of the object, is *D* = 0.32 for the triangular detector, and *D* = 0.52 for the rectangular detector.

**Figure 14:**
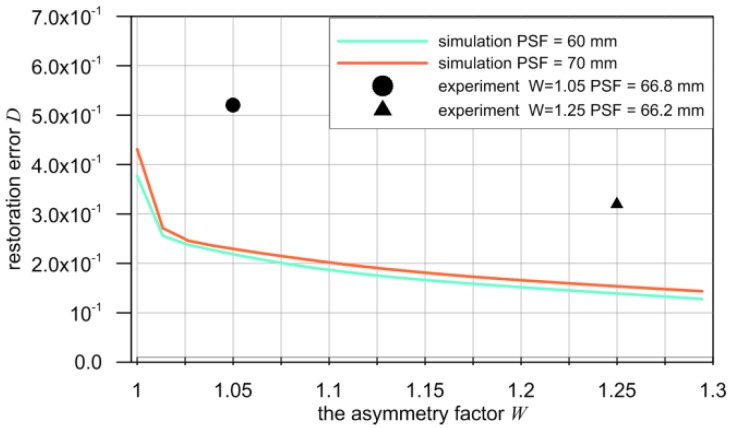
The comparison simulation results with the experiment.

A comparison of experimental and simulation results allows Figure 14. It shows a fragment of the characteristics shown in Figure 7 and two points determined experimentally. These points correspond to the two selected values of the PSF asymmetry factor *W* i.e., *W* = 1.05 and *W* = 1.25.

The differences between the results of the experiment and the simulation results may be due to several reasons. The PSF used in the experiment is not described by a function and its values were obtained in the identification process. For this reason, they are certainly subject to errors. In the simulation tests, PSF was modeled by compound shape of the two parts of the Gaussian curve. This shape differs from the shape of the PSF of the experiment, although the respective values of the factor *W* are the same. The simulation study was realized using a perfect digital convolution.

In fact, the convolution is only an approximation of the process performed during the scan of an object. Experimental studies, such as the identification of PSF and scanning of the object, are subject to the errors resulting from disturbances contained in the measuring signals. All this causes the observed difference in the obtained results, although, in the opinion of the authors, they are acceptable considering the complex phenomenon occurring during the scanning of the metal object by an eddy-current sensor. Please note that the model, based on convolution operations, is only an approximation of the phenomena occurring between the sensor and the object.

Both sensors used in experimental studies, in spite of various geometrical shapes, have very similar metrological characteristics. The PSF width of both detectors is comparable, which indicates that both the sensors have a similar averaging ability to that of the measurement signal. This is confirmed by the similar error value (10), used to evaluate the distance between the measured signal and the output signal of the sensor. On this basis, it is justified to claim that the observed improvement in the efficiency of the deconvolution algorithm is due to PSF asymmetry.

This thesis is also confirmed by the results of the simulation tests. The impact of the asymmetry factor *W* of the sensor’s PSF on the accuracy of the signal restoration, in the sense of error *D*, was investigated. In this study, however, any particular geometric shape of the sensor was not assumed.

Experimental tests have been carried out for the triangular sensor, for which the PSF asymmetry factor is equal *W* = 1.25. Tests for sensors with higher values of the asymmetry factor *W* were performed by means of computer simulation. Their results, shown in Figure 7, confirm the positive impact of the PSF asymmetry on the effectiveness of deconvolution and accuracy of the measuring signal restoration. Performing experimental tests with the sensors having a required degree of PSF asymmetry is however conditioned by the ability of designing such sensors. Currently, such a method is not known and its preparation requires further long-term experimental and simulation studies. The authors plan to undertake such research.

## 5. Conclusions

This paper presents the results of studies on an inductive loop used in systems classifying vehicles. A commonly used and widely accepted spatial convolution model for the classification of vehicle chassis was used in the study. One of the ways of increasing the spatial resolution of the magnetic signature obtained and the effectiveness of the classification process is a deconvolution operation. In many publications, numerical algorithms are presented to decrease the estimate error of the object shape signal. In this paper, a new approach leading to an improvement in the quality of the restoration signal obtained from an eddy-current sensor, is presented. This was achieved by shaping the sensor PSF. In the simulation studies presented, it is shown that even a slight PSF asymmetry results in a considerable decrease in the restoration error of the input signal. The results of experimental studies confirmed the assumptions of the simulations. Two inductive loop models were used in the experiments, with a symmetrical and asymmetrical shape with regard to the scanning direction. As a result of the application of an LS algorithm, a significantly smaller restoration error was obtained for the asymmetrical sensor. In light of these results, it appears justifiable to search for effective methods of designing eddy-current sensors with the PSF asymmetry.

Continuing research on the issue described in this paper, authors intend to develop a method allowing the design of eddy-current sensors with desired PSF asymmetry. The use of such sensors, in the form of an inductive loop, in the measurement systems of road traffic parameters, will allow a considerable increase in the information contained in the measuring signals, generated by the vehicles that pass over the sensor. The practical use of such sensors, in the authors’ opinion, will allow the efficient detection of the axles of trucks and cars, the estimation of the location of the axles relative to the characteristic points in the geometry of the vehicle (e.g., front and rear bumpers), and will allow the measurement of distances between axles, as well as detecting the presence of a trailer. As a consequence, the authors expect to significantly improve the effectiveness of vehicle classification using systems equipped only with inductive loops. Effectiveness could be significantly better than this, which is achieved by using the best systems, equipped with axle detectors, while the costs of such a system will be many times reduced.

## Figures and Tables

**Table 1:** PSF shape and its equivalent convolution matrix **H** form.

Type of System	PSF Shape	Convolution Matrix H Form
Symmetric PSF	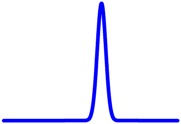	$\left[\begin{array}{ccccccc} h_{0} & h_{1} & h_{2} & 0\; & 0\; & \cdots & 0\\ h_{1} & h_{0} & h_{1} & h_{2} & 0\; & \ddots & \vdots \\ h_{2} & h_{1} & h_{0} & h_{1} & h_{2} & \ddots & \vdots \\ 0 & h_{2} & h_{1} & h_{0} & h_{1} & \ddots & 0\\ 0 & 0 & h_{2} & h_{1} & h_{0} & \ddots & h_{2}\\ \vdots & \ddots & \ddots & \ddots & \ddots & \ddots & h_{1}\\ 0 & \cdots & \cdots & 0 & h_{2} & h_{1} & h_{0} \end{array}\right]$
	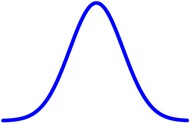	$\left[\begin{array}{ccccccc} h_{0} & h_{1} & h_{2} & h_{3} & h_{4} & \cdots & h_{M}\\ h_{1} & h_{0} & h_{1} & h_{2} & h_{3} & \ddots & \vdots \\ h_{2} & h_{1} & h_{0} & h_{1} & h_{2} & \ddots & h_{4}\\ h_{3} & h_{2} & h_{1} & h_{0} & h_{1} & \ddots & h_{3}\\ h_{4} & h_{3} & h_{2} & h_{1} & h_{0} & \ddots & h_{2}\\ \vdots & \ddots & \ddots & \ddots & \ddots & \ddots & h_{1}\\ h_{M} & \cdots & h_{4} & h_{3} & h_{2} & h_{1} & h_{0} \end{array}\right]$
Asymmetric	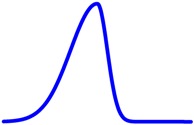	$\left[\begin{array}{ccccccc} h_{0} & h_{-1} & h_{-2} & 0 & 0 & \cdots & 0\\ h_{1} & h_{0} & h_{-1} & h_{-2} & 0 & \ddots & \vdots \\ h_{2} & h_{1} & h_{0} & h_{-1} & h_{-2} & \ddots & 0\\ h_{3} & h_{2} & h_{1} & h_{0} & h_{-1} & \ddots & 0\\ h_{4} & h_{3} & h_{2} & h_{1} & h_{0} & \ddots & h_{-2}\\ \vdots & \ddots & \ddots & \ddots & \ddots & \ddots & h_{-1}\\ 0 & \cdots & h_{4} & h_{3} & h_{2} & h_{1} & h_{0} \end{array}\right]$
